# Evaluating the Efficacy of Siddha Pharmacotherapy for Acrochordon: A Case Report

**DOI:** 10.7759/cureus.76391

**Published:** 2024-12-25

**Authors:** Saravanasingh Karan Chand Mohan Singh, Ramamurthy Murugan, Nithyamala I, Ethel Shiny S, V Gowri

**Affiliations:** 1 Department of Udal Koorugal, National Institute of Siddha, Chennai, IND; 2 Department of Noi Naadal, National Institute of Siddha, Chennai, IND; 3 Department of Gunapadam, National Institute of Siddha, Chennai, IND; 4 Department of Gunapadam, National Institute of Siddha, Tambaram Sanatorium, Chennai, IND; 5 Department of Gunapadam, Maria Siddha Medical College and Hospital, Thiruvattar, IND

**Keywords:** acrochordon, external application, pachaieruvai, siddha medicine, skin tag

## Abstract

Skin tags, medically known as acrochordons, are harmless growths of the epidermis that commonly develop in areas where the skin folds, such as the neck, armpits, or groin. While usually asymptomatic, these lesions can cause discomfort from rubbing or cosmetic issues. They are more prevalent in middle-aged and older individuals and are often correlated with conditions such as obesity, diabetes, and insulin resistance. The need for more specific treatment options for skin tags poses a significant challenge for healthcare providers, especially since the current methods, including electrodesiccation, laser therapy, cryotherapy, and surgical removal, are often expensive. However, Siddha medicine offers a potential alternative and cost-effective approach.

This report involves a 40-year-old male with a hefty, stalk-like, soft, flesh-toned, painless, movable, single-skin tag that had persisted for six years. Dermatological examination revealed a prominent acrochordon on the anterolateral aspect of the upper right thigh, measuring approximately 8 cm in diameter. Treatment involved applying the Siddha medicine Pachaieruvai externally for five consecutive days. During this period, the patient experienced mild pain, burning, slight redness, and localized swelling at the application site. These temporary symptoms lasted about an hour during and after each application but subsided within 24 hours after the application of coconut oil. The discomfort, redness, and swelling observed during the intervention were considered positive indicators of lesion regression. A follow-up assessment two weeks post-treatment showed significant clinical improvement. No adverse effects or recurrence were reported at subsequent follow-ups, suggesting that Siddha pharmacotherapy can be a viable alternative treatment for acrochordons.

## Introduction

Acrochordons, also known as fibroepithelial polyps or skin tags, are harmless skin growths characterized by their pedunculated, soft, flesh-colored, painless, and movable nature. These lesions generally pose no significant health risks, are usually asymptomatic, and require no medical treatment unless they cause aesthetic concerns or discomfort. They commonly appear in areas such as the neck (cervical), armpits (axillary), and groin (inguinal) [1] and have been associated with various conditions such as diabetes mellitus, obesity, hyperlipidemia, insulin resistance, acromegaly, and mechanical friction [2]. 

Individuals with acrochordons often have high BMI, elevated total cholesterol, triglycerides, low-density lipoprotein cholesterol (LDL-C) levels, and decreased high-density lipoprotein cholesterol (HDL-C) levels [3]. Human papillomavirus (HPV) has been identified in approximately 80% of skin tags, predominantly subtypes 6 and 11, suggesting a potential role in their formation, although the exact mechanism remains unclear [4,5]. Skin tags affect approximately 25-30% of the general population, particularly middle-aged and older individuals [6]. A strong correlation exists between acrochordons and obesity, with 74% of patients being either obese or overweight, and 24% meeting the criteria for metabolic syndrome [7].

Acrochordons are typically diagnosed through clinical examination, and physical assessment is sufficient for identification [8]. The treatment options include cryotherapy, mechanical devices, and surgical excision, each with its own effectiveness, risks, and side effects. Cryotherapy, particularly using devices such as the Pixie® Skin Tag cryogenic pen, has shown high success rates in fully removing lesions with a single application. Mechanical devices, such as adhesive patches, offer a noninvasive approach with minimal discomfort. Surgical methods, including snipping and the use of tissue forceps, provide rapid and effective removal [9,10,11].

This case report explores the potential of Siddha medicine in acrochordon management, specifically Pachaieruvai, as an efficient and economical alternative treatment. Siddha pharmacotherapy is a distinctive part of the Siddha system in its use of specially processed metals and minerals, like mercury, silver, arsenic, lead, and sulfur, in treating infectious diseases without side effects. It excels in providing effective therapy for conditions such as psoriasis, rheumatic disorders, chronic liver disorders, benign prostate hypertrophy, bleeding piles, peptic ulcers, and various dermatological disorders.

## Case presentation

A 40-year-old male presented to our outpatient clinic with a single, hefty, soft, skin-colored growth on the upper right thigh that had been present for the past six years. The growth was painless, movable, and attached to a small stalk. The dermatological assessment identified a significant skin tag on the anterolateral region of the upper right thigh, measuring approximately 8 cm in diameter (Video 1). The patient reported no pain or itching but noted friction on the fabric. He had no history of high blood pressure, diabetes, or other major health issues.

**Video 1:** Skin tag before treatment

Diagnosis

General and systemic examinations were normal. The lesion was diagnosed as a skin tag (acrochordon) based on the clinical presentation.

Management

The patient opted for removing the growth due to persistent irritation caused by friction against clothing that resulted in significant discomfort. The patient had not undergone any treatment before the intervention. However, he later chose the traditional Siddha medicine, Pachaieruvai, which was applied topically over the stalk for five consecutive days (Video 2). During treatment, the patient experienced mild pain, a burning sensation, slight redness, and localized swelling at the application site. These symptoms lasted about an hour during and after each application but were temporary and subsided within 24 hours of using coconut oil (Video 3). The discomfort, redness, and swelling observed during the intervention were considered positive indicators of lesion regression. Ultimately, the treatment destroyed the growth after 10 days (Video 4).

**Video 2:** Applying medicine over the stalk of the skin tag

**Video 3:** Skin tag three days after treatment

**Video 4:** Skin tag 10 days after treatment

Follow-up

The patient was reassessed after two weeks, with no signs of infection or recurrence. The lesion site had healed well. During the three-month follow-up after the completion of therapy, there were no signs of adverse effects or lesion recurrence, indicating both the efficacy and safety of the applied treatment regimen.

## Discussion

This report provides an initial exploration of treating acrochordon with Pachaieruvai, an age-old treatment rooted in Siddha medicinal traditions, as outlined in the Siddha texts. Pachaieruvai comprises Vellaipadanam (arsenic trioxide), Aridharam (arsenic trisulfide), Thurusu (copper sulfate), Karchunnam (calcium carbonate), and Kungiliyam (resin from Shorea robusta). Research has indicated that Vellaipaadanam, commonly recognized as arsenic trioxide and an essential element of Pachaieruvai, exhibits remarkable anti-glioma and antiviral effects. Evidence suggests that it curtails the growth of glioma cells while enhancing programmed cell death [12].

Arsenic trioxide (As_2_O_3_) notably activates apoptotic pathways in HPV16 DNA-immortalized human cervical epithelial cells (HCE16/3 cells) [13]. Aritharam, or arsenic trisulphide, exhibits cytotoxic characteristics by thwarting the emergence and expansion of solid tumors [14]. Thurusu, identified as copper sulfate, displays cytotoxic traits that hinder tumor development and possesses antiviral properties [15,16]. It triggers cytotoxic reactions by generating reactive oxygen species (ROS) [17]. Karchunnam, also known as calcium carbonate, exerts cytotoxic effects [18]. Similarly, red Kungiliyam, a resin derived from Shorea robusta, has cytotoxic capabilities that obstruct cellular growth [19]. Studies have shown that the Shorea robusta extract can inhibit the growth of cancer cells. For example, robustic acid derivatives have shown potent cytotoxicity against various cancer cell lines, including HL-60 (human leukemia ) and HepG2 (human hepatoblastoma) [20].

Mode of action

The inhibition of cellular proliferation by the constituents of Pachaieruvai can be attributed to mechanisms that promote cellular apoptosis, which likely played a crucial role in the effectiveness of the treatment. Additionally, the formulation may possess antiviral properties, potentially exerting therapeutic effects against HPV, thereby contributing to the overall regression of the lesion.

Treatment benefits

The treatment led to successful destruction of the large, movable skin tag and the patient was satisfied with the outcome.

Limitations of the study

Case studies focus on individual patients, limiting the ability to generalize the findings to larger populations. Although case studies can identify potential links between causes and outcomes, they cannot establish definitive cause-and-effect relationships. The absence of controlled trials and broad observational studies makes it difficult to determine the specific factors that influence skin tag progression. Owing to their limited scope, case studies may not fully represent the diverse manifestations or treatment responses of skin tags. Moreover, they often emphasize rare or dramatic cases, leading to potential bias in the literature, which can distort perceptions of the frequency and progression of skin tags. Despite these limitations, case studies remain useful for learning about treatments, generating research ideas, and illustrating the complexities of skin tag management in real-world scenarios. However, these findings should be interpreted cautiously and complemented with data from other research studies to gain a comprehensive understanding of skin tags.

## Conclusions

This report explores the use of Siddha methodology to treat acrochordon. The findings suggest that Siddha pharmacotherapy is highly effective in managing acrochordons. Integrating Pachaieruvai into this approach offers several advantages, including cost-effectiveness, time efficiency, lower dosage requirements, and targeted and localized therapeutic effects. Additionally, its economic feasibility makes it a compelling option in the therapeutic domain. Adopting a holistic healing strategy associated with traditional Siddha methods, addressing the patient's issues and concerns, could prove invaluable for treating acrochordon cases and ensuring the absence of recurrence. This case study presents a practical and economically viable approach to managing the quotidian challenges of Acrochordon, thereby enhancing the comprehension of the medical and socioeconomic aspects of its treatment. Furthermore, extensive clinical studies are imperative to gain deeper insights into these mechanisms. This represents a significant advancement in adopting a research-driven approach within a healthcare framework.
